# Effects of Upconversion Nanoparticles on Polymerase Chain Reaction

**DOI:** 10.1371/journal.pone.0073408

**Published:** 2013-09-05

**Authors:** Sang-Hyun Hwang, Su-Gyeong Im, Sang Soo Hah, Vu Thanh Cong, Eun Jeong Lee, Yeon-Su Lee, Geon Kook Lee, Do-Hoon Lee, Sang Jun Son

**Affiliations:** 1 Department of Laboratory Medicine, Center for Diagnostic Oncology, Research Institute and Hospital, National Cancer Center, Goyang-si, Republic of Korea; 2 Department of Chemistry and Research Institute for Basic Sciences, Kyung Hee University, Seoul, Republic of Korea; 3 Department of Chemistry, Gachon University, Seongnam, Gyeonggi, Republic of Korea; 4 Cancer Genomics Branch, Research Institute, National Cancer Center, Goyang-si, Republic of Korea; 5 Center for Lung Cancer, National Cancer Center Hospital, National Cancer Center, Goyang-si, Republic of Korea; 6 Lung Cancer Branch, Research Institute, National Cancer Center, Goyang-si, Republic of Korea; University of Houston, United States of America

## Abstract

Nanoparticles (NPs) are attractive materials owing to their physical and electrochemical properties, which make them extremely useful in diagnostic applications. Photon upconversion is the phenomenon where high-energy photons are emitted upon excitation of low-energy photons. Nucleic acids detection based on upconversion nanoparticles (UCNPs), which display a high signal-to-noise ratio and no photobleaching, has been widely applied. We evaluated whether UCNPs can improve polymerase chain reaction (PCR) specificity and affect PCR amplification. The effects of UCNPs with a diameter size of 40, 70, and 250 nm were evaluated using 3 PCR kits (AccuPower PCR PreMix, AmpliTaq Gold 360 Master Mix, and HotStarTaq Plus Master Mix) and 3 real-time PCR kits (AccuPower GreenStar qPCR PreMix, SYBR Green PCR Master Mix, and QuantiTect SYBR Green PCR Kit). Quantum dots were used for comparison with the UCNPs. In the presence of an appropriate concentration of UCNPs, PCR specificity was optimized. UCNPs of 40-nm size improved PCR specificity more effectively than did UCNPs sized 70 or 250 nm. As the size and concentrations of the UCNPs were increased, PCR amplification was more severely inhibited. At lower annealing temperatures (25°C–45°C), addition of the 40 nm UCNP (1 µg/µL) to the PCR reagent produced specific PCR products without nonspecific sequence amplification. Therefore, UCNPs of different sizes, with different DNA polymerases used in the commercial kits, showed different inhibitory effects on PCR amplification. These results demonstrate that optimization of UCNPs, added to reaction mixtures at appropriate concentrations, can improve PCR specificity. However, the mechanism underlining UCNPs effect on PCR remains unclear and will require further investigation.

## Introduction

Polymerase chain reaction (PCR) techniques have been in widespread use in areas that require DNA/RNA detection, such as diagnosis and forensic analysis. Improving the PCR yield and specificity is important for developing more efficient molecular diagnostic tests. Some organic chemicals like formamide and amides, added to the reaction mixture, can also enhance PCR amplification [1], [2].

Increasingly, nanoparticles (NPs) have become attractive materials owing to their physical and electrochemical properties, which make them extremely useful in the diagnostic applications for DNA [3], RNA [4], and protein [5]. Of special interest are quantum dots (QDs), gold nanoparticles, and upconversion nanoparticles (UCNPs). QDs are highly fluorescent, and in comparison with organic dyes are more bright and stable against photobleaching [6]. Gold nanoparticles show easily tuned physical properties, including unique optical properties, robustness, and high surface areas, making them ideal candidates for developing biomarker platforms [7].

Photon upconversion is the phenomenon where high-energy photons are emitted upon the excitation of low-energy photons. This is achieved through multiphoton processes [8]. There have been reports of the use of photon UCNPs in such applications as biosensing, imaging, and drug delivering [9]. Based on UCNPs, which display a high signal-to-noise ratio and no photobleaching, the DNA sensor demonstrates high sensitivity and specificity [10].

The evaluation of the effect of these nanoparticles on PCRs is important, because many PCR-based nucleic acid detection methods using nanoparticles have been reported, and the paradigm for current molecular diagnostic methods is shifting from conventional PCR to a rapid and homogeneous method with no separation step, such as real-time PCR [11]. Recently, gold nanoparticles and QDs have been reported to affect PCR amplification [12], [13], [14]. Li et al. [12] reported that adding gold nanoparticles to a PCR could avoid the production of nonspecific amplification products at lower annealing temperatures, and gold nanoparticles can improve the efficiency of PCRs [15]. QDs were reported to improve the PCR specificity through interaction with *Taq* polymerase [16].

Although UCNPs have been successfully used for ultrasensitive biological detection, especially DNA detection [17], there has been no research to evaluate the effect of UCNPs on the PCR specificity and amplification. Thus, we evaluated whether the UCNPs can improve the PCR specificity and affect the PCR amplification efficiency in the similar ways that QDs and gold nanoparticles do. We characterized the effect of UCNPs on the PCR using several commercial DNA polymerases, for the potential application of UCNPs in one-step nucleic acid detection in molecular diagnosis.

## Materials and Methods

### 1. DNA Template, UCNPs, and QDs

Genomic DNA was isolated from soybeans using the DNeasy Plant Mini Kit (Qiagen, Valencia, CA, USA), following the manufacturer’s instruction. UCNPs of different sizes (40, 70, and 250 nm) were prepared. Avidin-conjugated UCNPs with a size of approximately 40 nm were purchased from Sigma-Aldrich (upconversion nanocrystals UCP 545; Sigma-Aldrich, St. Louis, MO, USA), and others (70 and 300 nm) were prepared according to the literature [18], [19], [20]. The UCNPs were diluted in a 1× Tris-acetate-EDTA buffer solution at pH 8.5. Quantum dots with a size of approximately 30 nm (Qdot 545 ITK streptavidin conjugate; Invitrogen, Carlsbad, CA, USA) were used for comparison with the UCNPs.

### 2. Preparation of UCNPs of 70 and 250 nm Sizes

All chemicals were purchased from Sigma-Aldrich. Yttrium(III) chloride, ytterbium(III) chloride, erbium(III) chloride, sodium hydroxide, ammonium fluoride, oleic acid, 1-octadecene, toluene, ammonium hydroxide, tetraethyl orthosilicate, and (3-aminopropyl)triethoxysilane (APTES) were used as supplied, without further purification.

#### 2.1 Synthesis of UCNPs

UCNPs were prepared following a previously reported protocol [18], [19], [20]. Methanol solutions of yttrium(III) chloride (2 mL, 0.4 M), ytterbium(III) chloride (0.45 mL, 0.4 M), and erbium(III) chloride (0.05 mL, 0.4 M) were prepared and added to a 100 mL 3-head flask containing 6 mL of oleic acid and 15 mL of 1-octadecene. After the solution was heated to 140°C to dissolve the powder for 30 min, a clear yellow solution was obtained and allowed to cool to room temperature. Subsequently, 10 mL of a methanol solution of ammonium fluoride (0.15 g) and sodium hydroxide (0.1 g) was added dropwise to the flask, with constant stirring, for 10 min and maintained for another 30 min. The solution was heated to 110°C to evaporate the methanol and water. The temperature was then increased to 315°C, and the solution was kept for 40 min under argon. UCNPs were collected and purified by centrifugation (5000 rpm, 10 min) with a mixed solution of cyclohexane/ethanol (volume ratio, 1∶5). Finally, the UCNPs were re-dispersed in 10 mL of toluene.

#### 2.2 Silica coating and APTES modification

**Figure 1:**
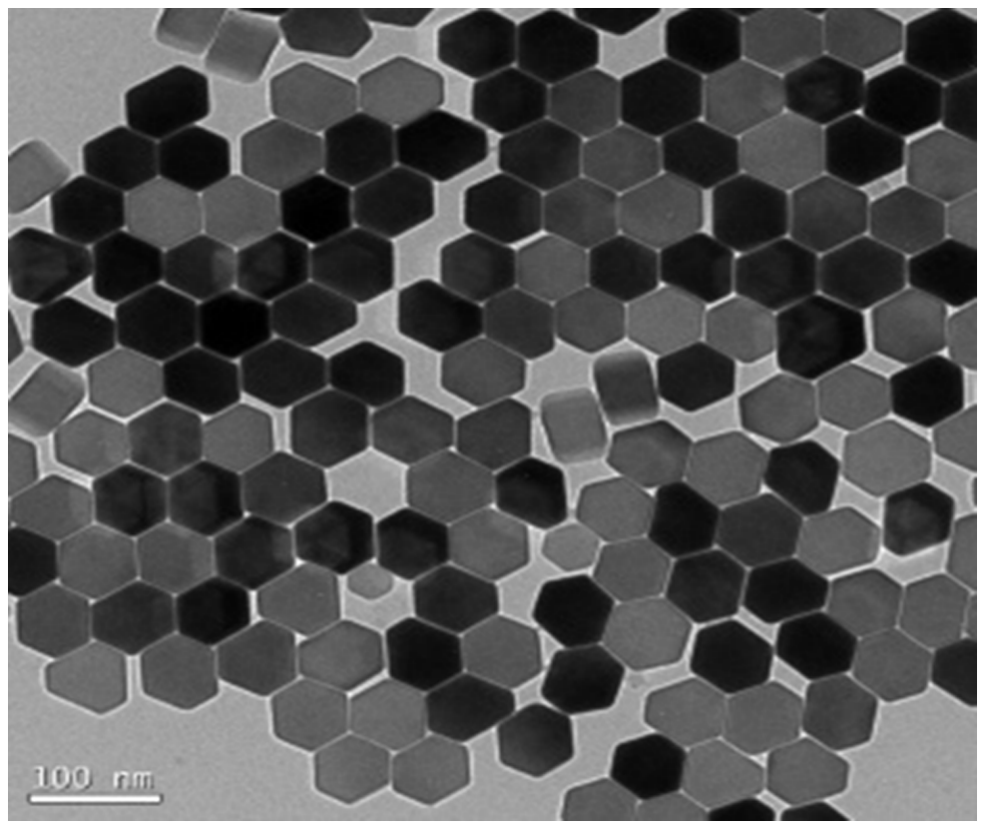
Transmission electron microscopy images of 70 nm bare upconversion nanoparticles (UCNPs).

To a solution of UCNP (20 mg) in 3-propanol (60 mL), 22.5 mL of aqueous ammonium hydroxide solution (2.5% [w/v]) was added. Subsequently, tetraethyl orthosilicate (25 µL) in 20 mL of 3-propanol was added dropwise to the reaction mixture for 15 min, with vigorous stirring, and the solution was allowed to stand for 4 h to yield silica-coated UCNPs. These UCNPs were used for the APTES modification without further purification. For the APTES modification, a solution of APTES (0.2 mL) in 2-propanol (30 mL) was added dropwise to the silica-coated reaction mixture. The reaction was kept for 1 h at room temperature. Finally, the amine-modified UCNPs were collected by centrifugation (2000 rpm, 10 min) and purified by washing 3 times with deionized water (Figure 1).

### 3. PCR Amplification

The forward primer 5′-GTGCGATCATACCAGCRYTAATGAACCGG-3′ and reverse primer 5′-GAGGTGCAACACGAGGACTTCCCAGGAGG-3′ were used to amplify a 120 bp 5S rRNA repeat [16]. Three commercial PCR master mixes, including the AccuPower PCR PreMix (Bioneer, Daejeon, Korea), AmpliTaq Gold 360 Master Mix (Applied Biosystems, Foster City, CA, USA), and HotStarTaq Plus Master Mix (Qiagen, Valencia, CA, USA), were used for the evaluation of the UCNPs effects on the PCR. *Top* DNA polymerase, used in the AccuPower PCR PreMix, was a modified form of a recombinant DNA polymerase, originally isolated from *Thermus thermophilus*. The AmpliTaq Gold DNA Polymerase in the AmpliTaq Gold 360 Master Mix, and the HotStarTaq Plus DNA Polymerase in the HotStarTaq Plus Master Mix (Qiagen) were recombinant forms modified from the *Thermus aquaticus* (*Taq*) DNA polymerase.

Aliquots of the UCNPs solutions were added to the PCR mixtures to achieve appropriate concentrations. DNA amplifications were performed in a final volume of 20 µl containing 0.5 µM of primers and 10 ng of DNA template. The final reaction volume was completed to 20 µl using sterilized double distilled water. A GeneAmp 9700 thermal cycler (Applied Biosystems, Foster City, CA, USA) was used for the DNA amplification. For the PCR, the conditions were as follows [16]: 4 min at 94°C; 35 cycles at 94°C for 30 s, 55°C for 30 s, and 72°C for 1 min; and, finally, 72°C for 10 min. In order to identify the effects of the UCNPs at lower annealing temperatures, PCR amplification was performed at different temperatures ranging from 25°C to 60°C by using the AccuPower PCR PreMix (Bioneer). The PCR products were analyzed by electrophoresis using a 2.0% agarose gel.

### 4. Real-Time PCR

SYBR Green real-time PCR with the same primers and conditions was performed for evaluating the effects of the UCNPs on PCR amplification efficiency. All experiments were performed in duplicate. Three commercial real-time PCR master mix kits, including the AccuPower GreenStar qPCR PreMix (Bioneer), SYBR Green PCR Master Mix (Applied Biosystems), and QuantiTect SYBR Green PCR Kit (Qiagen), were used. In this study, the coefficient of variation was 5.92%.

## Results

### Effects of UCNPs on PCR Specificity and Amplification Efficiency

**Figure 2:**
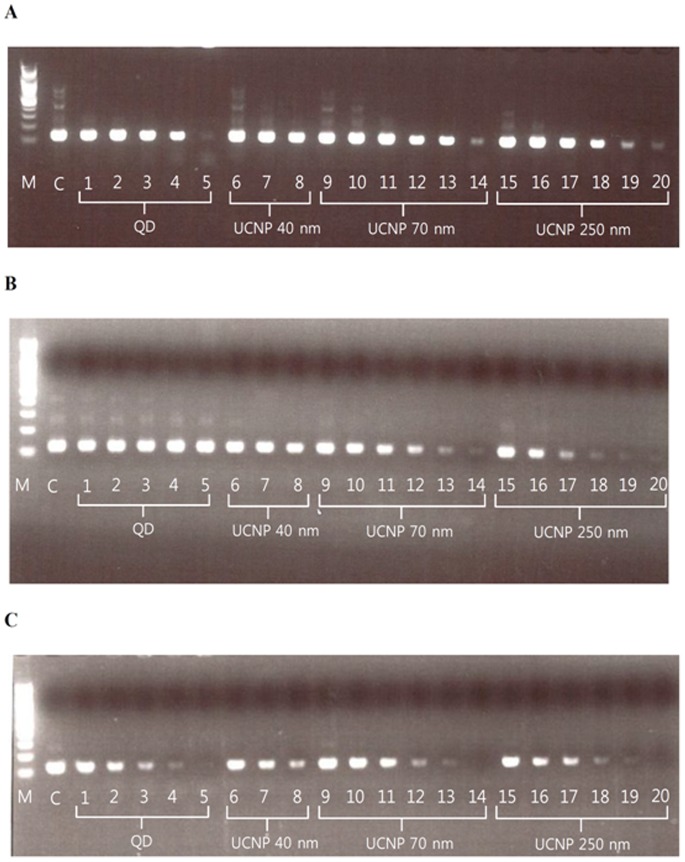
Effects of different concentrations and sizes of UCNPs on PCR amplification specificity. Three commercial real-time PCR master mixes were applied to determine the effects of different concentrations and sizes of UCNPs on the specificity of PCR amplification of a 120 bp 5S rDNA fragment from soybean genomic DNA, at an annealing temperature of 50°C. (**A**) AccuPower PCR PreMix with *Top* Polymerase (Bioneer). (**B**) AmpliTaq Gold 360 Master Mix with AmpliTaq Gold DNA Polymerase (Applied Biosystems). (**C**) HotStarTaq Plus Master Mix with HotStarTaq DNA Polymerase (Qiagen). Lane M: DNA marker; lane C: template without UCNPs; lanes 1∼5∶5, 7.5, 10, 15, and 30 nM of QDs, respectively; lanes 6∼8∶0.5, 0.75, and 1.0 µg/µL of 40-nm-sized UCNPs, respectively; lanes 9∼14∶1×( = 2.4×10^5^ particles/µL), 10×, 15×, 20×, 25×, and 30× of 70-nm-sized UCNPs, respectively; lanes 15∼20∶1×, 10×, 15×, 20×, 25×, and 30× of 250-nm-sized UCNPs, respectively.

The specificity of the PCR was improved at an appropriate concentration of UCNPs (Figure 2). UCNPs with a size of 40 nm improved the specificity of the PCR more effectively than did UCNPs with a size of 70 or 250 nm. As the concentrations of the UCNPs increased, the PCR amplification was more severely inhibited.

With the AccuPower PCR PreMix with *Top* Polymerase, the optimal concentrations of UCNPs for improving the PCR specificity were 1.0 µg/µL for the 40-nm-sized UCNPs, and 20×(1× = 2.4×10^5^ particles/µL) and 15× for the 70- and 250-nm-sized UCNPs, respectively (Figure 2A), and the C_T_ value was 13.92±0.06, 14.18±0.36, and 14.67±0.05, respectively, in the real-time PCR (AccuPower GreenStar qPCR PreMix; 13.45±0.15 without UCNPs; Table 1).

**Table 1:** Effects of UCNPs on PCR amplification efficiency, using 3 commercial real-time PCR master mixes.

UCNPs size	Concentration	AccuPower GreenStar qPCRPreMix (Bioneer, C_T_(mean±sd))	QuantiTect SYBR Green Kit(Qiagen, C_T_ (mean±sd))	SYBR Green PCR Master Mix(Applied Biosystems, C_T_(mean±sd))
5 s DNA	Reference (no UCNPs)	13.45±0.15	13.24±0.07	12.22±0.05
40 nm	1x (0.5 ug/ul)	14.23±0.13	13.44±0.07	12.76±0.15
	1.5x	14.08±0.07	13.19±0.04	13.70±1.50
	2x	13.92±0.06	13.26±0.39	15.63±0.66
70 nm	1x (2.4×10^5^ particles/ul)	13.27±0.01	13.19±0.03	12.36±0.15
	10x	13.74±0.16	13.50±0.05	13.14±0.23
	15x	13.91±0.23	13.70±0.06	13.68±0.51
	20x	14.18±0.36	14.03±0.23	13.87±0.11
	25x	14.18±0.14	14.77±0.43	16.15±1.42
	30x	16.83	15.02±0.26	UD*
250 nm	1x (2.4×10^5^ particles/ul)	13.55±0.53	13.35±0.09	12.33±0.04
	10x	14.14±0.09	14.29±0.02	13.26±0.35
	15x	14.67±0.05	14.69±0.16	13.64±0.27
	20x	15.94v.05	16.31±0.01	15.50±0.59
	25x	17.92	16.51±0.32	UD*
	30x	UD*	18.35±0.53	UD*

* UD; undetected.

With the AmpliTaq Gold 360 Master Mix with AmpliTaq Gold DNA Polymerase, the optimal concentrations of UCNPs for improving the PCR specificity were 0.75 µg/µL for the 40-nm-sized UCNPs, and 10× and 15× for the 70- and 250-nm-sized UCNPs, respectively (Figure 2B), and the C_T_ value was 13.70±1.50, 13.14±0.23, and 13.64±0.27, respectively (SYBR Green PCR Master Mix; 12.22±0.05 without UCNPs; Table 1). On the other hand, the nonspecific bands were not suppressed by the addition of QDs when the AmpliTaq Gold DNA Polymerase in AmpliTaq Gold 360 Master Mix was used for the PCR (Figure 2B). Complete PCR inhibition was observed when 25× to 30× UCNPs of 70 and 250 nm were added to only the AmpliTaq Gold 360 Master Mix with AmpliTaq Gold DNA Polymerase.

With the Qiagen HotStarTaq Plus DNA Polymerase in the Qiagen QuantiTect SYBR Green PCR Kit, the optimal concentrations of UCNPs for improving the PCR specificity were 0.5 µg/µL for the 40-nm-sized UCNPs, and 10× and 1× for the 70- and 250-nm-sized UCNPs, respectively, with a C_T_ value of 13.44±0.07, 13.50±0.05, and 13.35±0.09, respectively (QuantiTect SYBR Green PCR Kit; 13.24±0.07 without UCNPs; Table 1).

### PCR Amplification at Low Annealing Temperature

**Figure 3:**
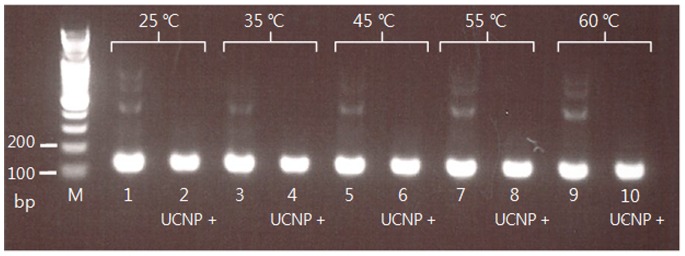
Effects of different annealing temperatures on PCR amplification specificity. A 120 bp 5S rDNA fragment from soybean genomic DNA was PCR-amplified with and without the use of a 40-nm-sized UCNP (1 µg/µL). Lane M: DNA marker; lanes 1, 2∶25°C; lanes 3, 4∶35°C; lanes 5, 6∶45°C; lanes 7, 8∶55°C; lanes 9, 10∶60°C. Lanes 1, 3, 5, 7, and 9 show the results of PCRs performed in the absence of UCNPs; lanes 2, 4, 6, 8, and 10 show the results of PCRs performed in the presence of UCNPs (1 µg/µL).

At annealing temperatures ranging from 25°C to 60°C, specific PCR products were obtained, without nonspecific amplification, by addition of the 40 nm UCNP (1 µg/µL) to the PCR master mix (Figure 3).

## Discussion

We demonstrated that the specificity of the PCR was effectively improved and no obvious effect on PCR amplification was seen at an appropriate concentration of the UCNPs of different sizes. Even at low annealing temperatures, addition of the UCNPs to the PCR reagent produced specific PCR products, without any nonspecific sequence amplification. Even on longer sized amplicons (>1 kb), we observed increased specificity of PCR product after adding UCNPs in PCR mixture for amplifying human leukocyte antigen genes (Figure S1). When the concentration was increased, however, PCR amplification was inhibited. Thus, the concentration and size of the UCNPs are important for PCR amplification.

UCNPs of different sizes, with different types of DNA polymerases in the several commercial kits, showed different inhibitory effects on PCR amplification. UCNPs of 40 nm had less of an inhibitory effect on the PCR than those of larger sizes (70 or 250 nm). This phenomenon was similar to the gold nanoparticles. Wan et al. [14] reported that gold nanoparticles could interact with *Taq* polymerase to inhibit the PCR, and the size of the gold nanoparticles had an impact on the PCR inhibitory effect. Vu et al. [13] showed that the inhibitory effect caused by gold nanoparticles, regardless of specificity, was due to the surface interaction of the *Taq* polymerase and gold nanoparticles, rather than heat transfer enhancement.

Although the size-dependent effect of QDs on PCRs has not been fully evaluated so far, QDs of 2.5 and 4.1 nm also showed a PCR inhibitory effect [16]. The size of the QDs used in our study was about 30 nm. It was also found that QDs could increase the specificity of the PCR at different annealing temperatures [21]. Bovine serum albumin (BSA) can interact with gold nanoparticles and QDs. The PCR inhibition caused by gold nanoparticles and QDs can be reversed by adding more BSA to the reaction solution [14], [16]. However, the UCNPs did not show the interaction with BSA in this study (Figure S2).

The DNA polymerase in the Bioneer PCR master mix was *Top* Polymerase, which is a modified form of a recombinant DNA polymerase, originally isolated from *T. thermophilus*, and the AmpliTaq Gold DNA Polymerase and HotStarTaq DNA Polymerase are modified from *Taq*. The DNA-dependent DNA polymerase activity of the *T. thermophilus* polymerase is broadly similar to that of the *Taq* polymerase [22]. Because the different commercial kits showed different inhibitory effects of the UCNPs on PCR amplification in this study, the effects of the PCR specificity and amplification efficiency using UCNPs could depend on the types of DNA polymerases used and the constituents in the PCR reagents.

These observed effects of UCNPs on the PCR are also seen in other studies with nanoparticles. Therefore, we speculated that UCNPs might have similar effects on the PCR owing to the interaction with DNA polymerases. One of possible mechanism could be the effective electrostatic interaction between the PCR component and nanoparticles for enhancing PCR [23]. Our surface of UCNPs was positively charged due to amine-modified UCNPs for functionalization. However, the exact mechanism of the UCNP effect on the PCR still requires further investigation. Moreover, we did not evaluate whether UCNPs would reduce the occurrence of primer-dimers or primer self-annealing.

In current molecular diagnoses, a homogeneous detection method such as real-time PCR is one of the most popular techniques, as it has the advantages of having no need for gels, sample manipulation, and separation steps. These gold nanoparticles, QDs, and UCNPs, through PCRs, would be widely applied to DNA detection [17]. UCNPs are composed of rare earth lanthanide elements embedded in a crystal and are capable of converting excitation light of lower energy (infrared light, 980 nm) to a higher energy emission. UCNPs are an emerging fluorescent nanoparticle, exhibiting several advantages over conventional fluorophores, such as a large anti-Stokes effect, high signal-to-noise ratio, and superior photostability [24]. These solution-based assays using UCNPs were applied in nucleic acid-based detection, such as for sickle cell anemia [9] and ankylosing spondylitis [25], with high sensitivity. Our study on the effects of UCNPs on PCR amplification and specificity would be helpful for developing future UCNP-based nucleic acid detection systems.

In this study, the specificity of PCRs at low annealing temperatures could also be effectively improved by UCNPs (Figure 3). At annealing temperatures from 25°C to 60°C, positive effects on PCR amplification were observed, similar to the effect of QDs [16]. Annealing temperature is one of the most important parameters that need adjustment in PCRs, and a relatively high annealing temperature is almost always required for a routine PCR. In this aspect, the result implies that UCNPs can simplify the optimization of PCRs.

Although different kinds of surface-modified UCNPs have not yet been fully evaluated, UCNPs may have similar effects on PCRs, regardless of surface modifications, like other nanoparticles, as was repeatedly reported [15], [16]. The adsorption of proteins onto nanoparticles can affect their structure and function, giving rise to either beneficial effects or unpredictable and potentially undesirable effects [26]. Therefore, the surface interaction between UCNPs and DNA polymerase should be characterized.

In conclusion, this study is the first ever to demonstrate that UCNPs at appropriate concentrations can increase the specificity of PCR amplification, even under low annealing temperatures, whereas they inhibit PCR at concentrations above a threshold value. The size of the UCNPs has an impact on the PCR inhibitory effect. This study provides initial data from the use of UCNPs in PCR-based nucleic acid detection for biomedical applications, such as molecular diagnosis.

## Supporting Information

Figure S1 To confirm the effect of UCNPs on the specificity of PCR for different targets, the human leukocyte antigen gene (HLA-A, -B, and –C) alleles were amplified by PCR primers using the AlleleSEQR class I kit (Abbott Molecular Inc., Des Plaines, IL) as manufacturer’s instruction. These kits are designed to provide high resolution identification of alleles of human HLA-A, -B, and –C genes. For HLA-typing PCR, the conditions are as follows: 1) 10 min at 95 C 2) 35 cycles at 96 C for 20 s, 60 C for 30 s and 72 C for 3 min, and finally 3) 72 C for 10 min. PCR products were analyzed by electrophoresis using a 2.0% agarose gel. Lane 1: HLA-A PCR product (arrow, just under 2 kb), Lane 2: HLA-B (arrow head, specific product), Lane 3: HLA-C (arrow head, specific product) without UCNP and Lane 4, 5, 6: HLA-A, -B, and –C with UCNPs, respectively. Non-specific bands (smaller sized amplicons) were suppressed by UCNPs.
(DOC)

Figure S2 The effect of the different concentrations of BSA on PCR amplification specificity of a 120 bp 5S rDNA fragment from soybean genomic DNA at an annealing temperature of 65°C with 1 ug/ul of UCNPs: lane M: DNA marker; lane N: negative control; C: 0 ug/ml; lane 1∶25 ug/ml; lane 2∶50 ug/ml; lane 3∶100 ug/ml; lane 4∶250 ug/ml; lane 5∶500 ug/ml; lane 6∶1000 ug/ml; lane 7∶2500 ug/ml of BSA (left). The effect of the different concentrations of BSA on PCR amplification specificity without UCNPs (right).
(DOC)
