# Supplementary material for: Effects of Upconversion Nanoparticles on Polymerase Chain Reaction
Source: PLoS One. 2013 Sep 5;8(9):e73408. doi: 10.1371/journal.pone.0073408 (PMC3764166; doi:10.1371/journal.pone.0073408)
Supplement: Figure S1 — To confirm the effect of UCNPs on the specificity of PCR for different targets, the human leukocyte antigen gene (HLA-A, -B, and –C) alleles were amplified by PCR primers using the AlleleSEQR class I kit (Abbott Molecular Inc., Des Plaines, IL) as manufacturer’s instruction. These kits are designed to provide high resolution identification of alleles of human HLA-A, -B, and –C genes. For HLA-typing PCR, the conditions are as follows: 1) 10 min at 95 C 2) 35 cycles at 96 C for 20 s, 60 C for 30 s and 72 C for 3 min, and finally 3) 72 C for 10 min. PCR products were analyzed by electrophoresis using a 2.0% agarose gel. Lane 1: HLA-A PCR product (arrow, just under 2 kb), Lane 2: HLA-B (arrow head, specific product), Lane 3: HLA-C (arrow head, specific product) without UCNP and Lane 4, 5, 6: HLA-A, -B, and –C with UCNPs, respectively. Non-specific bands (smaller sized amplicons) were suppressed by UCNPs. (DOC) [file pone.0073408.s001.doc]

Supplementary Fig. S1
