# Supplementary material for: Effects of Upconversion Nanoparticles on Polymerase Chain Reaction
Source: PLoS One. 2013 Sep 5;8(9):e73408. doi: 10.1371/journal.pone.0073408 (PMC3764166; doi:10.1371/journal.pone.0073408)
Supplement: Figure S2 — The effect of the different concentrations of BSA on PCR amplification specificity of a 120 bp 5S rDNA fragment from soybean genomic DNA at an annealing temperature of 65°C with 1 ug/ul of UCNPs: lane M: DNA marker; lane N: negative control; C: 0 ug/ml; lane 1∶25 ug/ml; lane 2∶50 ug/ml; lane 3∶100 ug/ml; lane 4∶250 ug/ml; lane 5∶500 ug/ml; lane 6∶1000 ug/ml; lane 7∶2500 ug/ml of BSA (left). The effect of the different concentrations of BSA on PCR amplification specificity without UCNPs (right). (DOC) [file pone.0073408.s002.doc]

Supplementary Fig. S2
